# Frequency distribution of TATA Box and extension sequences on human promoters

**DOI:** 10.1186/1471-2105-7-S4-S2

**Published:** 2006-12-12

**Authors:** Wei Shi, Wanlei Zhou

**Affiliations:** 1 School of Engineering and Information Technology, Deakin University, 221 Burwood Hwy, Burwood, VIC 3125, Australia

## Abstract

**Background:**

TATA box is one of the most important transcription factor binding sites. But the exact sequences of TATA box are still not very clear.

**Results:**

In this study, we conduct a dedicated analysis on the frequency distribution of TATA Box and its extension sequences on human promoters. Sixteen TATA elements derived from the TATA Box motif, TATAWAWN, are classified into three distribution patterns: peak, bottom-peak, and bottom. Fourteen TATA extension sequences are predicted to be the new TATA Box elements due to their high motif factors, which indicate their statistical significance. Statistical analysis on the promoters of mice, zebrafish and drosophila melanogaster verifies seven of these elements. It is also observed that the distribution of TATA elements on the promoters of housekeeping genes are very similar with their distribution on the promoters of tissue specific genes in human.

**Conclusion:**

The dedicated statistical analysis on TATA box and its extension sequences yields new TATA elements. The statistical significance of these elements has been verified on random data sets by calculating their *p *values.

## Background

Transcription factor binding sites (TFBSs) play a very important role in the regulation of gene expressions. Much research has been conducted on the discovery of TFBSs using computational approaches. Most of this research tries to discover all kinds of TFBSs [1-5]. However, the specific category of TFBSs, for example TATA Box, has not been analyzed in enough depth. The methods developed in the literature are targeted at discovering TFBSs in the general sense; this however is not suitable for the discovery of the specific category of TFBSs. In this research, we focus solely on the TATA Box, which is one of the most important TFBSs. The TATA Box (also named the Goldberg-Hogness box after its discoverers) is the first core promoter element identified in eukaryotic protein-coding genes [6]. In addition to the TATA Box elements, their extension sequences will also be analyzed to determine their frequency distribution across the entire range of human promoters.

A TATA Box extension sequence is a short DNA sequence which consists of a TATA Box element and several bases flanking this element from either the left, or the right, or both sides. The analysis on TATA Box extension sequences will shed more insights on the mechanism of the binding between the TATA Binding Protein and the TATA Box found in gene promoters.

The frequency distribution of TATA elements and extension sequences are analyzed on six data sets of human promoters. Two of the data sets were downloaded from the USCS genome database: one includes 20647 human promoters 1000 bp upstream from Transcription Start Sites (TSSs), and the other includes 17516 human promoters 2000 bp upstream from TSSs. All of the promoter sequences in these two sets have previously been aligned to the TSSs. It is also important to note that all of the repeated promoters in each of these two sets were deleted (repeats happen when multiple mRNA correspond to one same gene). Therefore after the adjustment the final numbers of promoters in these two sets are 17407 and 15491 respectively. And S_1000 _and S_2000 _denote these two sets respectively. The other four data sets are derived from these two sets by further classifying genes into housekeeping genes and tissue-specific genes. Lists of housekeeping genes and tissue-specific genes are collected from references [7-10]. S_hk1000 _and S_hk2000 _denote the sets of promoters of housekeeping genes with length 1000 and 2000 respectively, and S_ts1000 _and S_ts2000 _denote the sets of promoters of tissue-specific genes with length 1000 and 2000 respectively. The numbers of promoters in these four sets are 855, 910, 1267, and 1220, respectively.

## Results

Promoters which have been aligned to their TSSs are divided into a number of bins, each of which contains 20 bp from each gene. We investigate the frequency distribution of the single nucleotides, TATA elements and TATA extension sequences on different sets of promoters. And we compare our findings from the human promoters with the findings from promoters of mice, zebrafish and drosophila melanogaster.

### Frequency distribution of A, T, G and C in human promoters

**Figure 1:**
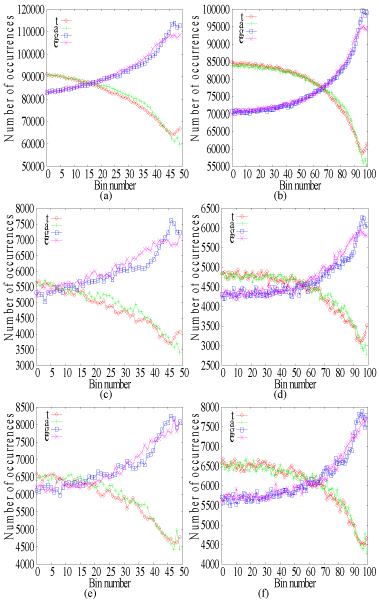
**Frequency distribution of single nucleotides (A, T, G, C) on six data sets**. Figure 1 shows the frequency of the four single nucleotides (A, T, G, C) on six different data sets: (a)S_1000_. (b)S_2000 _(c)S_hk1000 _(d)S_hk2000 _(e)S_ts1000 _and (f)S_ts2000_. In each figure, x axis is the bin number and y axis is the number of occurrences of a single nucleotide in a bin. G/C content is shown to be much higher than A/T content at the location close to TSSs in all figures. But there is a small increase of A/T content at the location where TATA Box resides(the second closest bin to TSSs). This can help explain why there is a TATA Box in a area where the majority of bases are G and C. At the 5' end of promoters far from TSSs, A/T content is observed to be higher than G/C content. And little difference of frequency of single nucleotides is observed between housekeeping genes and tissue specific genes when comparing Figure 1(c) and Figure 1(e) and comparing Figure 1(d) and Figure 1(f).

First of all, we determine the distribution of each of the four single bases A, T, G and C in each of six data sets. The results are shown in Figure 1. Bin 49 is the bin closest to TSSs. A/T have lower abundance at the location close to TSSs, while G/C have much higher abundance at that location. A, T, G and C show the same abundance at the location of around 700 bp upstream from TSSs (around 35 bins upstream from TSSs). From Figure 1(a) and 1(b), it is observed that the frequency distribution of A, T, G and C on the data set S_1000 _is very similar with their frequency distribution on S_2000_. Their frequency distribution on the promoters of housekeeping genes is almost the same as that on the promoters of tissue-specific genes as shown in Figure 1(c) to 1(f), except the slightly different locations where A, T, G and C has got the same abundance in these two data sets.

### Frequency distribution of TATA elements

**Figure 2:**
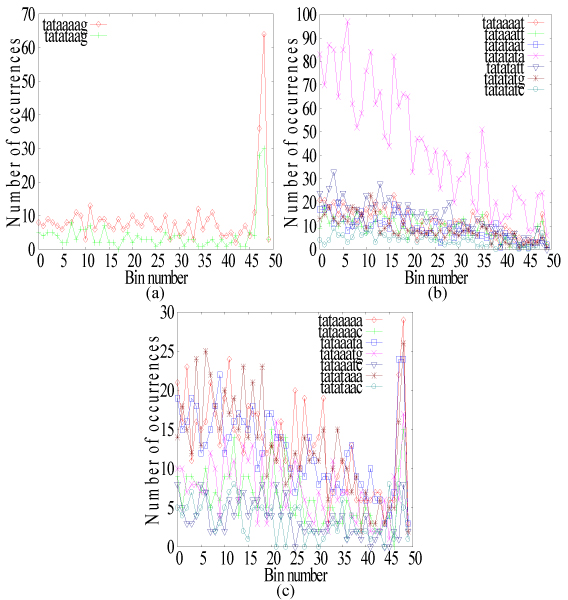
**Three patterns of frequency distribution of TATA elements on the data set S**_**1000**_. Sixteen TATA elements are classified into three frequency patterns: (a)Peak pattern: including two TATA elements(TATAAAAG and TATATAAG) which show very high abundance at the location close to TSSs, but do not show any abundance at other locations; (b)Bottom pattern: including seven TATA elements which show decreasing abundance from 5' end of promoters to TSSs; (c)Bottom-peak pattern: including seven TATA elements which show decreasing abundance from 5'end of promoters to near TSSs, but there is strong abundance at the location close to TSSs which is higher than the other locations.

TATA Box contains sixteen elements. We investigate the frequency distribution of all these sixteen elements on the data set S_1000 _and calculate their Motif Factors (MFs, see methods). Two elements (TATAAAAG and TATATAAG) show very high abundance at the location close to TSSs, but do not show any abundance at other locations (peak pattern). Figure 2(a) show the frequency distribution of these two TATA elements. The maximal occurrence number of TATAAAAG is 64, which appears at bin 48 (-20~-40 bp upstream from TSSs). The maximal occurrence number of TATATAAG is 30, which appears at bin 48 also. The MFs of TATAAAAG and TATATAAG are 19(*p *< 1e-16) and 9(*p *< 1e-16) respectively.

Seven TATA elements show decreasing abundance from 5' end of promoters to TSSs (bottom pattern). This is shown in Figure 2(b). The maximal occurrence numbers of these elements appear in the remote 5' end of the promoters, rather than at the location close to TSSs. In these elements, TATATATA has the biggest occurrence number (97) which appears at bin 6. It is also observed that the occurrence number of TATATATA is much larger than any other TATA elements. TATATATA's minimal occurrence number is 6, which appears at bin 49 (the bin closest to TSSs). TATATATA's total number of occurrences is 2200, which is much larger than the total occurrence numbers of any other TATA Box element as well.

The frequency distribution of the remaining seven TATA elements is shown in Figure 2(c). These elements show decreasing abundance from 5'end of promoters to near TSSs, but there is strong abundance at the location close to TSSs which is higher than the other locations (bottom-peak pattern). The maximal occurrence numbers of these elements appear at the location close to TSSs. All these elements except TATATAAC get their maximal occurrence numbers in the second closest bin to TSSs (bin 48). The maximal occurrence number of TATATAAC appears at bin 47. The general trend for the frequency distribution of these seven elements is: at first the occurrence numbers markedly drop at the approximate location of bin 40 from 5' end to TSSs (around 200 bases upstream from TSSs), then a sharp increase occurs at the location of bin 48, and finally the occurrence numbers drop again in the last bin. Bin 48 is the location where the TATA Box is supposed to reside.

In data set S_2000_, TATAAAAG and TATATAAG show similar frequency distribution pattern. They have got very high abundance at the location close to TSSs (figures not shown). The maximal occurrence numbers of these two elements are 57 and 26 respectively, these figures are slightly smaller than their maximal occurrence numbers in S_1000_. The MFs of TATAAAAG and TATATAAG in S_2000 _are 12.3 and 7.3 respectively.

The seven elements which show the bottom pattern of frequency distribution in S_1000 _show the similar pattern in S_2000_. TATATATA still has the largest total occurrence number in S_2000_, with an average number of occurrences of 50.8, a maximal occurrence number of 111 which appears at bin 42, and a minimal occurrence number of 4 which appears at bin 99 (the bin closest to TSSs for the data set S_2000_).

It is noted that seven elements which show the bottom-peak pattern of frequency distribution in S_1000 _show a different pattern in S_2000_. They do not show any abundance at the location close to TSSs at all in S_2000 _(figures not shown). The maximal occurrence numbers of these seven elements appear at the location at least 1120 bases upstream from TSSs. This outcome implies that these seven elements might not be the real TATA elements, or that perhaps different binding mechanisms are applied to these elements.

**Figure 3:**
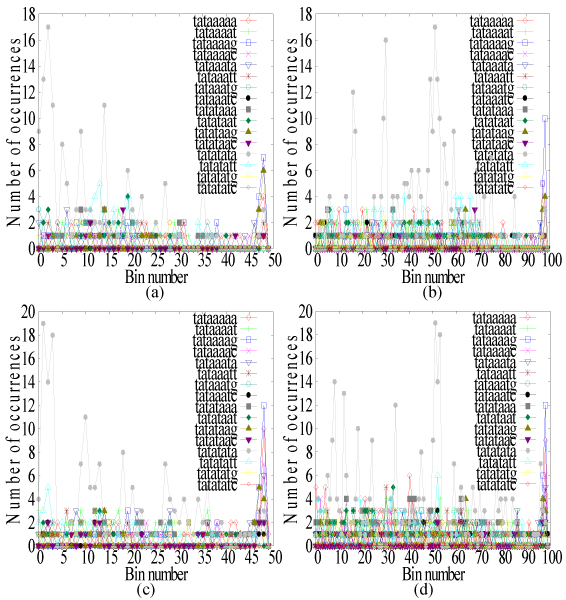
**Frequency Distribution of sixteen TATA Box elements on promoters of housekeeping genes and tissue specific genes**. Frequency distribution of sixteen TATA elements is observed on four data sets: (a)S_hk1000 _(b) S_hk2000 _(c)S_ts1000 _and (d)S_ts2000_. These four data sets represent 1000 bp long promoters of housekeeping genes, 2000 bp long promoters of housekeeping genes, 1000 bp long promoters of tissue specific genes and 2000 bp long promoters of tissue specific genes respectively. The frequency distribution of sixteen TATA elements on housekeeping genes is very similar with their frequency distribution on tissue specific genes by comparing Figure 3(a) with Figure 3(c) and comparing Figure 3(b) with Figure 3(d).

For data set S_hk1000 _and S_hk2000_, all sixteen TATA elements have very low occurrence numbers as shown in Figure 3(a) and Figure 3(b). The majority of the occurrence numbers are 0, 1 or 2. It is also observed that the distribution of TATATATA is again much higher than the other elements. The frequency distribution of sixteen TATA elements in S_ts1000 _is shown in Figure 3(c), which is similar with their frequency distribution in S_hk1000 _(as shown in Figure 3(a)). Likewise, the frequency distribution of these elements in S_ts2000 _(as shown in Figure 3(d)) is similar with their frequency distribution in S_hk2000 _(as shown in Figure 3(b)).

### Frequency distribution of TATA extension sequences

We investigate the frequency distribution of TATA extension sequences which extend TATA elements from either the left, or the right, or the both sides on data sets S_1000 _and S_2000_. We do not however investigate their distribution on the remaining data sets, because these extension sequences have very small numbers of occurrences within them and therefore it would have been very difficult to mine meaningful information from such low frequency distributions.

**Figure 4:**
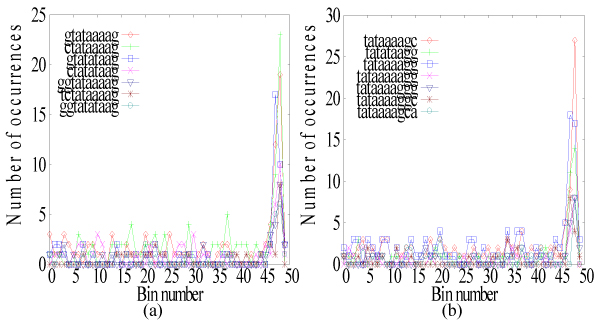
**Frequency distribution of fourteen TATA Box extension sequences on S**_**1000**_. Fourteen TATA extension sequences are found to show very high abundance at the location close to TSSs. Seven of them extend TATA elements from the left side as shown in Figure 4(a), and seven others from the right side as shown in Figure 4(b). Thirteen out of these fourteen sequences extend from TATAAAAG or TATATAAG. Calculation of *p *values for these fourteen sequences demonstrates that their high motif factors are not obtained by chance.

The seven TATA extension sequences which extend TATA elements from the left have very high occurrence numbers at the location close to TSSs in S_1000 _as depicted in Figure 4(a). These sequences extend from TATAAAAG or TATATAAG. Figure 4(b) shows the seven TATA extension sequences which extend TATA Box elements from the right. These extension sequences have very high occurrence numbers at the location close to TSSs in S_1000 _as well. The majority of these sequences also extend from TATAAAAG and TATATAAG.

The TATA extension sequences which extend the TATA elements from both sides do not show high abundance at the location close to TSSs.

The observed distribution patterns of the fourteen TATA extension sequences shown in Figure 4(a) and Figure 4(b) are very similar with their distribution patterns on S_2000 _(figures not shown).

TATAAAAG and TATATAAG, from which the fourteen TATA extension sequences mainly extend, show the strongest peaks at the location close to TSSs. They also have the largest MFs amongst all TATA Box elements. The MFs of these two TATA elements and the fourteen TATA extension sequences are not less than 6 in both S_1000 _and S_2000 _as shown in Table 1. They have got very low *p *values which demonstrate their high MFs are not obtained by chance.

### Frequency distribution of TATA elements and TATA extension sequences on other organisms

It is assumed that biologically significant TATA elements and TATA extension sequences will be conserved during the course of evolution. Therefore, we select several organisms which have different evolution distances from human including mice, zebrafish, and drosophila melanogaster, to verify the TATA elements and TATA extension sequences with high motif factors discovered in the above sections.

**Figure 5:**
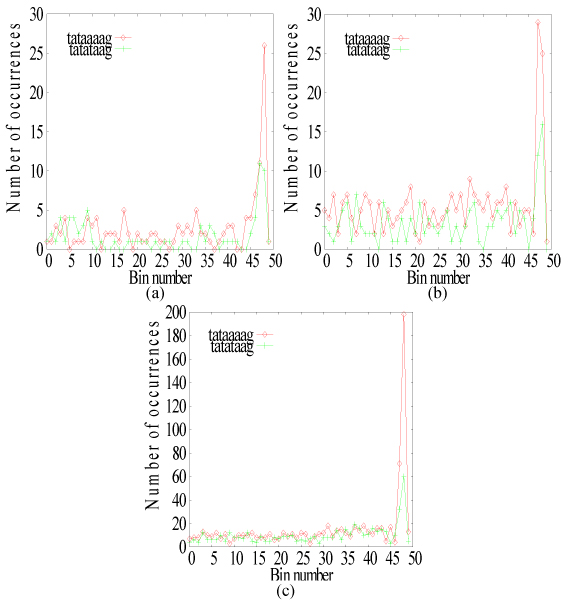
**Frequency distribution of TATAAAAG and TATATAAG on three other organisms**. TATAAAAG and TATATAAG are the two TATA elements which show peak pattern of frequency distribution in human promoters. The investigation on their frequency distribution on other three organisms shows the same pattern. Figure 5(a), (b) and (c) show their frequency distribution on the gene promoters of mice, zebrafish and drosophila melanogaster respectively.

Figure 5 shows the frequency distribution of TATAAAAG and TATATAAG in the gene promoters of mice, zebrafish, and drosophila melanogaster. TATAAAAG shows the strongest peak amongst all the TATA elements in the three organisms as observed in the human promoters. Its motif factor in Drosophila is the largest amongst the four organisms at 37.4. Its motif factor in human is the second largest at 19. The percentage of human promoters containing TATAAAAG is 2.5%, which is the lowest percentage among the four organisms. Zebrafish has the largest percentage of promoters containing this TATA element (6.4%), however, its motif factor is the smallest.

The MF of TATATAAG in human is the largest among the four organisms. However, the percentage of human promoters containing this element is the lowest (1.2%). As with the TATAAAAG, the zebrafish receives the highest percentage of promoters containing TATATAAG. Hence from the perspective of evolution, there is a decrease in the percentage of gene promoters containing TATAAAAG or TATATAAG. The investigation on the other TATA elements leads to the same conclusion.

Because of the extremely limited number of occurrences of TATA extension sequences extending TATA elements by more than one base in mice, zebrafish and drosophila melanogaster, we only calculate the frequency distributions of TATA sequences with one base extension on the promoters of the three organisms and compare them with their distribution in human promoters.

A TATA extension sequence will be regarded as a conserved sequence if its motif factor in each organism is not less than 2. Seven conserved TATA extension sequences are discovered from this research as shown in Table 2. It is also observed that all of the seven sequences show strong peaks at the location close to TSSs in mice, zebrafish and drosophila melanogaster besides human. These seven sequences extend from the same two TATA elements as we mentioned before: TATAAAAG and TATATAAG. In these seven extension sequences all the extension bases are either G or C. This is a reasonable occurrence for human promoters because the G/C content is much higher than the A/T content at the location where TATA Box resides.

The TATA extension sequence, TATAAAAG***G***, has the biggest motif factor amongst all TATA elements and TATA extension sequences in all four organisms (42). However, only 0.7 percent of human promoters contain this sequence, this is the lowest in the percentages of promoters containing this element in any of the four organisms. TATAAAAG***C ***has the largest motif factor in human (26). The percentage of human promoters containing this sequence is also the lowest in the percentages of promoters containing this element in all four organisms (0.6%).

The average percentage of promoters containing TATA extension sequences in human is lower than the average percentage in any of the other organisms, which can be observed from Table 2. And the drosophila has the highest average percentage of promoters containing TATA extension sequences. This suggests that the percentage of gene promoters containing TATA extension sequences is decreasing in the course of evolution. However, the seven TATA extension sequences shown in Table 2 are well conserved due to their high motif factors in each organism, which verifies the TATA extension sequences extending from some TATA elements by one base discovered by the statistical analysis on human promoters. The TATA extension sequences of two bases extension from some TATA elements are not examined here because of their very small number of occurrences in the bins of gene promoters of three other organisms.

## Discussion

The TATA Box is a T/A-rich sequence that is usually located 25–35 base pairs upstream of the TSSs. Recruitment of TBP and TBPassociated factors, all part of the TFIID complex, directs assembly of the pre-initiation complex (PIC), a highly regulated process that ensures precise initiation of transcription [11]. The consensus sequence of TATA Box is TATAWAWN [12].

It is interesting to note that single nucleotides (A, T, G and C) show similar distribution pattern in the promoters of three types of genes (housekeeping genes S_hk1000 _and S_hk2000_, tissue specific genes S_ts1000 _and S_ts2000_, and all genes S_1000 _and S_2000_). Figure 1 shows that the A/T content decreases from the remote 5' end of the promoters to TSSs. But there is a sudden increase of A/T content at the location close to the TSSs. We can use this to help explain why at this location there is a TATA Box which mainly consists of A and T. The G/C content increases from 5' end of the promoters to TSSs. At the location close to TSSs, the G/C content was much higher than the A/T content. Therefore TATA box should be easily identified at this location because of the very low A/T content in this area.

According to the early studies, TATA Box is strictly conserved and essential for transcription initiation from all protein-coding genes from yeast to man. However, the recent research suggests that the prevalence of the TATA Box diminished [6]. Suzuki et al indicates that TATA boxes are present in 32% of 1031 potential core promoters [13]. A genome wide analysis points out that the percentage of TATA-containing promoters is much lower than commonly recognized [11]. Our study shows that only 24% of 17407 human promoters of length 1000 contain TATA boxes. We also find that 27% of mice promoters, 74.4% of zebrafish promoters and 64.9% of drosophila promoters contain TATA Box.

It is observed that two TATA elements TATAAAAG and TATATAAG have extraordinarily higher abundance at the location close to TSSs than any other TATA elements. MFs of these two elements were 19(*p *< 1e-16) and 9(*p *< 1e-16) respectively, but MFs of other TATA elements range from only 1.3 to 3.1. TATATAAG is identified as the optimal TBP recognition sequence [14]. TATAAAAG, as asymmetrical TATA sequence, is bound by TBP in a polar manner in the TBP-DNA cocrystals [15,16]. The orientation of an asymmetrical TATA box will influence the transcription direction [17,18].

We find that those TATA extension sequences which have high MFs are mostly flanked by G and C. Bucher demonstrated that TATA Box is often flanked by G/C-rich sequences [19]. Of the fourteen TATA extension sequences identified from human promoters in this study, ***G***TATAAAAG is observed upstream the 5S rRNA genes of both 5S rDNA classes detected in O. niloticus [20]. Also the ***C***TATAAAAG is a conserved TATA motif [21].

In addition to these two TATA extension sequences, the other five TATA extension sequences which extend TATA elements by one base are verified by the comparative analysis on the promoters of human, mice, zebrafish and drosophila melanogaster (Table 2). We do not however investigate the frequency distribution of other TATA extension sequences which extend TATA elements by more than one base due to their very low occurrence numbers in mice, zebrafish and drosophila melanogaster.

## Conclusion

This paper uses a statistical approach to analyze the frequency distribution of TATA elements and TATA extension sequences on the promoters of human and three other organisms. The contributions of this work are three-fold: (1) Detailed analysis on the frequency distribution of TATA elements and TATA extension sequences on human promoters; (2) Comparison between the frequency distribution of TATA elements on the promoters of human housekeeping genes with their frequency distribution on the promoters of human tissue specific genes; (3) Comparison between the frequency distribution of TATA elements and TATA extension sequences on human promoters with their frequency distribution on the promoters of mice, zebrafish and drosophila melanogaster.

We suggest that the TATA extension sequences discovered as a result of this research are the potential candidates of new transcription factor binding sites due to their extraordinarily high statistical significance. Elucidation of the frequency distribution of TATA elements and TATA extension sequences on the promoters of human and other organisms will contribute to the better understanding of TATA Box and the determination of exact TATA binding sites.

## Methods

### Data Sets

Promoters of length 1000 for human, mice, zebrafish and drosophila melanogaster were downloaded from the UCSC Genome database (, human version May 2004, mice version June 2003, zebrafish version June 2004, and drosophila version April 2004). Repeated promoters were eliminated from the gene promoter set for each organism. Thus each gene has only one corresponding promoter in each of these data sets(the original data included redundant gene promoters which would bring noise to the statistical analysis. The redundancy is caused by the multiple mRNAs that one gene might have). The final numbers of promoters for human, mice, zebrafish and drosophila are 17407, 3465, 4248 and 12841 respectively.

Promoters of length 2000 for human were downloaded from USCS Genome database as well. After deleting repeated ones, we were left with 15491 promoters.

### Human housekeeping genes and tissue specific genes

Housekeeping genes and tissue specific genes were collected from the literature [6-9]. Those genes were determined by microarray experiments. After deleting the repeated genes found by different researches, we got 981 housekeeping genes and 1416 tissue specific genes for human. Based on the lists of housekeeping genes and tissue specific genes and the data sets containing gene promoters of length 1000 and 2000 downloaded from USCS genome database, we finally got 855 promoters of length 1000 for housekeeping genes, 910 promoters of length 2000 for housekeeping genes, 1267 promoters of length 1000 for tissue specific genes and 1220 promoters of length 2000 for tissue specific genes.

### Statistics of TATA elements and TATA extension sequences on gene promoters

Gene promoters of length 1000 are divided into 50 bins. Bins are numbered from 0 to 49. Bin 49 is the closest bin to TSSs. And gene promoters of 2000 are divided into 100 bins. Bin 99 is the closest bin to TSSs. Each bin contains a stretch of 20 bases from each promoter. For calculating the statistical significance of a TATA Box element or an extension sequence called *s*, we first calculate its occurrence number in each bin. This number is calculated as the sum of each occurrence number of *s *appearing in each promoter at that bin. For a bin *b *in a promoter *p*, *s *occurs in *b *if *s *is a substring of *p *and its first letter is located in *b*. If *s *falls on the bin boundary, the position of the first letter of *s *would determine which bin *s *belongs to. One promoter may be counted multiple times if there are multiple TATA elements or TATA extension sequences in that promoter. The motif factor (MF) of *s *is calculated as:



where *x*_*max *_is the maximal occurrence number in all bins.  is the median occurrence number of the sorted sequence of all occurrence numbers of *s*. And *sd *is the standard deviation which is calculated as:

*sd *= *x*_3 _- *x*_1_

where *x*_3 _and *x*_1 _are the third quartile and first quartile of *s*'s sorted sequence of occurrence numbers respectively.

MF describes how the peak of the frequency distribution of TATA elements and TATA extension sequences deviates from the median value.

### Calculation of *P *value

*P *values are calculated for TATA elements and TATA extension sequences in Table 1 to test whether their high motif factors are obtained by chance or not. 1000 sets of random promoters for human were generated using a seventh-order Markov model [3]. The motif factor of each TATA element and TATA extension sequence in Table 1 was determined on each set of random promoters. Distribution of the motif factor is assumed to comply with normal distribution. After calculating the mean value and standard deviation of the motif factor for each TATA element and TATA extension sequence, we used the R package [22] to calculate the probability that the values of the motif factor observed on 1000 sets of random promoters are equal to or greater than the value observed in the real set of human promoters. The smaller the *P *value, the more nonrandom the result.

## Authors' contributions

WS conceived the study, implemented the algorithm and drafted the manuscript. WZ supervised the project. All authors contributed to, read and approved the final manuscript.

## Figures and Tables

**Table 1:** Motif factors and *p *values of TATAAAAG and TATATAAG and their extension sequences on S_1000 _and S_2000_.

Sequences	S_1000_	S_2000_	*p*
TATAAAAG	19	12.3	<1e-16
***G***TATAAAAG	18	8.5	<1e-16
***GG***TATAAAAG	8	8	2.3e-10
***C***TATAAAAG	11	10	<1e-16
***TC***TATAAAAG	8	6	1.6e-11
TATAAAAG***C ***	26	21	<1e-16
TATAAAAG***CA ***	7	9	8.6e-8
TATAAAAG***G***	8.3	7.2	1.9e-15
TATAAAAG***GC ***	8	12	1.4e-12
TATAAAAG***GG ***	8	9	2.0e-12
TATATAAG	9	7.3	<1e-16
***G***TATATAAG	17	15	<1e-16
***GG***TATATAAG	6	8	<1e-16
***C***TATATAAG	9	8	8.3e-14
TATATAAG***G***	13.5	11	<1e-16
TATAAAAA***GG***	8	8	4.0e-12

TATA extension sequences which are statistically significant mainly extend from two TATA elements: TATAAAAG and TATATAAG. Table 1 gives the motif factors and *p *values for these two TATA elements and fourteen TATA extension sequences. *P *values are calculated based on the human promoters of length 1000 bp. In Table 1, bases of italic bold font are the extension bases.

**Table 2:** Summary of statistical analysis on TATAAAAG and TATATAAG and their extension sequences on gene promoters of four organisms.

	Human		Mice		Zebrafish		Drosophila	
	
	MF	%	MF	%	MF	%	MF	%
TATAAAAG	19	2.5	12	3.8	8	6.4	37.4	5.9
***G***TATAAAAG	18	0.5	6	0.8	13	1.4	40.3	1.5
TATAAAAG***G***	8.3	0.7	5	1.0	9	1.1	42	1.0
***C***TATAAAAG	11	0.5	9	1.2	9	1.0	34.5	1.4
TATAAAAG***C***	26	0.6	9	1.1	6.5	1.8	30.8	1.9
TATATAAG	9	1.2	5	2.3	4.3	4.0	8.7	3.8
***G***TATATAAG	17	0.3	3	0.6	8	0.6	7	0.6
TATATAAG***G***	13.5	0.3	5	0.8	2	0.4	6.5	0.6
***C***TATATAAG	9	0.3	7	0.8	6	0.6	15	0.8

TATAAAAG and TATATAAG and seven of their extension sequences are found to be statistically significant in all four organisms including human, mice, zebrafish and drosophila melanogaster (their motif factors are equal to or greater than 2 in each organism). These TATA elements and TATA extension sequences are believed to be conserved in the course of evolution. Seven TATA extension sequences in this table are also included in Table 1. In this table, bases of bold italic font denote the extension bases, MF denotes Motif factor and % denotes the percentage of promoters which contain the corresponding sequence in the first column.
